# Family quality‐of‐life burden in chronic spontaneous urticaria: A multicentre study

**DOI:** 10.1111/jdv.70290

**Published:** 2026-01-05

**Authors:** Beatrice Martinez Zugaib Abdalla, Roberta Fachini Jardim Criado, Kanokvalai Kulthanan, Emek Kocatürk, Ivan Cherrez‐Ojeda, Ana Maria Giménez‐Arnau, Marcus Maurer, Luis Felipe Ensina, Jonathan A. Bernstein, Michihiro Hide, Sergio Duarte Dortas Junior, Solange Oliveira Rodrigues Valle, Gabriela Dias, Paraskevi Xepapadak, Priyanshi Dangi, Indrashis Podder, Vesna Trajkova, Natasa Teovska Mitrevska, Iman Hamed Nasr, Bushra Al Hinai, Joanna Bartosińska, Dorota Krasowska, Paulina Szczepanik‐Kułak, Hilal Gungor, Deniz Ozceker, Kübra Çiçek, Esen Özkaya, Yasemin Erdem, Mücahit Ergün, Mustafa Tosun, Rabia Oztas Kara, Jie Li, Anastasia Allenova, Paulo Ricardo Criado

**Affiliations:** ^1^ Centro Universitário Faculdade de Medicina do ABC Santo André Brazil; ^2^ Siriraj Urticaria and Angioedema Center Bangkok Thailand; ^3^ Institute of Allergology, Charité – Universitätsmedizin Berlin Corporate Member of Freie Universität Berlin and Humboldt‐Universität zu Berlin Berlin Germany; ^4^ Fraunhofer Institute for Translational Medicine and Pharmacology ITMP Immunology and Allergology Berlin Germany; ^5^ Department of Dermatology Bahçeşehir University Istanbul Turkey; ^6^ Universidad de Especialidades Espiritu Santo Samborondon Ecuador; ^7^ Hospital del mar & Research Institute Barcelona Spain; ^8^ Federal University of São Paulo Unifesp São Paulo Brazil; ^9^ University of Cincinnati College of Medicine Cincinnati USA; ^10^ Hiroshima University Hiroshima Japan; ^11^ Clementino Fraga Filho University Hospital Rio de Janeiro Brazil; ^12^ Pedro Ernesto University Hospital Rio de Janeiro Brazil; ^13^ Panagiotis, and Aglaia Kyriakou Children's Hospital National and Kapodistrian University of Athens Athens Greece; ^14^ D Y Patil Hospital Navi Mumbai India; ^15^ College of Medicine & Sagore Dutta Hospital Kolkata India; ^16^ City General Hospital ‘September 8th’ – Skopje Skopje North Macedonia; ^17^ Re‐Medika Hospital Skopje North Macedonia; ^18^ The Royal Hospital Muscat Oman; ^19^ Medical University of Lublin Lublin Poland; ^20^ Health Sciences University Istanbul Turkey; ^21^ Hacettepe University Medicine Faculty Ankara Turkey; ^22^ İstanbul University İstanbul Faculty of Medicine, Department of Dermatology and Venereology İstanbul Turkey; ^23^ Sivas Cumhuriyet Üniversitesi Sivas Turkey; ^24^ Sakarya University Sakarya Turkey; ^25^ The Second Xiangya Hospital of Central South University Chang Sha Shi China; ^26^ Sechenov University Moscow Russia

**Keywords:** chronic spontaneous urticaria, family relations, health expenditures, health status indicators, health‐related quality of life, omalizumab, quality of life, sickness impact profile, skin disease, urticariaantihistamines

## Abstract

**Background:**

Chronic spontaneous urticaria (CSU) can cause psychosocial and quality of life burden on patients and their family members and caregivers. Despite its recognition as a debilitating disease, limited data exist regarding the impact of CSU on family members, hindering a comprehensive understanding of the disease's broader effects. This study aimed to assess how CSU affects the quality of life of family members who support patients in their daily challenges by applying the Family Dermatology Life Quality Index (FDLQI) questionnaire across multiple countries.

**Methods:**

A cross‐sectional, multicentre and international study conducted between January and December 2024 in Urticaria Centres of Reference and Excellence (UCARE) centres located in several countries including Brazil, China, Ecuador, Greece, India, Oman, Poland, Russia, Thailand, Turkey, Peru and North Macedonia. Statistical analyses, including non‐parametric tests and multiple regression models, were employed to explore associations between disease severity/control and family burden.

**Results:**

Poorly controlled CSU significantly deteriorated family members' quality of life, particularly in emotional, physical and social domains. Higher disease severity and lower disease control scores were associated with increased stress, greater caregiving burden and elevated health expenditures. In opposition to family relations, older age and longer time since diagnosis mitigate negative impacts, while insufficient treatment regimens exacerbated them.

**Conclusions:**

Inadequate control of CSU amplifies the burden on families, underscoring the need for effective and supportive care strategies.

## Plain Language Summary

Chronic spontaneous urticaria (CSU) is a skin condition that causes itchy hives, swelling and discomfort lasting more than 6 weeks. It affects up to 1% of people worldwide, especially women aged 30–50. Although not life‐threatening, it can severely impact daily life and emotional well‐being. This international cross‐sectional study was conducted in 12 countries across three continents, from January to December 2024 at Urticaria Centres of Reference and Excellence (UCARE). The study aimed to investigate how CSU affects the family members of patients, focusing on emotional, physical, social and financial impacts. A total of 1187 CSU patients and their respective family members completed questionnaires, including the Family Dermatology Life Quality Index (FDLQI), and physicians also provided clinical data on disease severity and control. Results showed that uncontrolled CSU was linked to more household responsibilities and emotional stress for family members, often disrupting their work and personal routines. In contrast, strong family support and effective treatment were associated with lower family burden. Early diagnosis, appropriate treatment and continuous follow‐up can reduce this burden and improve quality of life for both patients and their families.

## Graphical abstract

Poor control of chronic spontaneous urticaria (CSU) significantly worsens family members' quality of life, especially in emotional, physical, and social domains. Higher disease severity was linked to increased caregiving burden and household expenditures, highlighting the need for family‐centred CSU care and support across diverse global populations.

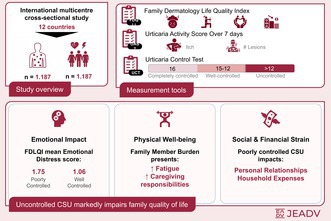

**Why was the study undertaken?**

- To better understand the broader impact of Chronic Spontaneous Urticaria (CSU) on the families and caregivers of patients. While the burden of CSU on patients is well‐documented, the effect on those in close contact with the patient has not been identified.

**What does this study add?**

- Higher disease severity and lower disease control scores are associated with increased stress, caregiving burden, and household expenses.
- These are new insights that show the need for broader evaluation and support mechanisms in managing chronic urticaria.

**What are the implications of this study for disease understanding and/or clinical care?**

- To highlight the importance of early diagnosis, treatment and follow‐up that could enhance well‐being, personal and family quality of life.

## INTRODUCTION

Chronic spontaneous urticaria (CSU)^1^, ^2^, ^3^, ^4^, ^5^ is associated with a substantial burden and significantly impacts patients' health‐related quality of life (HRQoL), often posing treatment challenges.^1^, ^4^ Patients may experience reduced productivity and higher rates of psychological comorbidities.^6^, ^7^ Over 30% of CSU patients present high Dermatology Life Quality Index (DLQI) scores, primarily due to the sudden appearance of lesions and pruritus.^1^ Recurrent symptom onset, fatigue, pain, anxiety and depression also burden CSU patients.^8^

Although often considered a benign and self‐limiting condition, CSU is increasingly recognized as a debilitating disease, particularly when symptoms persist.^9^, ^10^ It can^4^, ^10^ have an HRQoL impact comparable or exceeding that of moderate‐to‐severe psoriasis, atopic dermatitis (AD), asthma and severe coronary artery disease requiring bypass grafting.^1^, ^4^, ^6^, ^7^, ^10^, ^11^, ^12^, ^13^, ^14^

Chronic skin diseases can also burden families and caregivers. The ‘*Greater Patient’* concept acknowledges this by assessing the families' quality‐of‐life (QoL).^15^ Some studies have reported increased emotional distress and household expenditures^16^

Skin conditions such as vitiligo, pemphigus vulgaris and atopic dermatitis have been shown to negatively impact family QoL, particularly when conditions are severe or long lasting.^17^, ^18^, ^19^ The Family Dermatology Life Quality Index (FDLQI) is a validated instrument for dermatological conditions and is appropriate for CSU, as it captures key aspects of family burden such as emotional distress, caregiving demands and the impact of chronic pruritus and disease unpredictability^20^, ^21^

The pathophysiological complexity of CSU, involving autoimmune mechanisms, limits therapeutic responses, prolongs disease activity and adds to patient and family suffering.^22^, ^23^ Despite international guidelines advocating a stepwise approach and recognized efficacy of medications such as omalizumab, many patients (~30%) remain inadequately controlled, exacerbating the psychosocial impact of CSU.^24^, ^25^

Most studies focus on patient outcomes, with limited data on how disease activity and control impacts family members. Furthermore, cultural, geographic and sociodemographic factors may influence perception, treatment access and coping strategies, complicating generalization of findings.^26^, ^27^

### Objectives

This study aims to employ the FDLQI questionnaire to evaluate how CSU affects families of patients on their relationships, their emotional, physical, social and economic burden, as well as involvement in daily disease management across several countries.

## METHODS

### Study design

A cross‐sectional, multicentre, international study was conducted from January to December 2024 across several centres within the Urticaria Centres of Reference and Excellence (UCARE) network. Data were collected from centres located in Brazil, China, Ecuador, Greece, India, Oman, Poland, Russia, Thailand, Turkey, Peru and North Macedonia, representing diverse populations across three continents.

### Population and inclusion criteria

Participants included family members and caregivers of patients diagnosed with CSU who were under regular follow‐up at UCARE centres. This study received initial approval from Brazilian Ethics Committee (#4931797). Written informed consent was obtained from all participants prior to data collection.

Inclusion criteria encompassed individuals over 18 years of age, in the same household as the CSU patient, and possessing sufficient literacy to comprehend the informed consent form and questionnaires in their native language. Exclusion criteria included individuals not meeting these requirements or those who declined participation. All patients included in this study were under CSU treatment.

### Data collection

Data were gathered through self‐administered questionnaires completed by family members, supplemented by clinical information provided by the attending physicians responsible for the patients at each participating centre. The questionnaire comprised a demographic section with 10 items and the FDLQI.

The FDLQI is a validated 10‐item questionnaire that assesses the impact of a skin disease on the QoL of close family members. Total scores range from 0 to 30, with higher scores indicating greater impairment.

For clinical assessment, physicians completed two standardized instruments: the Urticaria Activity Score over 7 days (UAS7) and the Urticaria Control Test (UCT).

### Statistical analysis

Descriptive analyses summarized participant sociodemographic characteristics, utilizing absolute and relative frequencies. Data were stratified by participants' relationship to patients, and group comparisons used Pearson's chi‐squared test for categorical data or Fisher's exact test when appropriate. FDLQI dimensions were compared across CSU control levels using the Kruskal–Wallis test for UAS7 and the Mann–Whitney *U*‐test for UCT.

Three multiple regression analyses were conducted, with the FDLQI acting as the dependent variable in all models. The first model included demographic and clinical variables. The second and third models examined the relationships between FDLQI and UAS7, and between FDLQI and UCT, respectively.

All analyses were conducted using R software (version 4.4.1) through the RStudio interface, with significance level set at *p* < 0.05.^28^, ^29^

Further methodological details are provided as supplementary content.

## RESULTS

### Demographic characteristics of the study population

Between January and December 2024, a total of 2374 participants were enrolled across multiple international UCARE centres,^30^ comprising 1187 CSU patients and 1187 accompanying family members or caregivers. Immediate family members represented the predominant group.

Participants were predominantly female (57.5%) with a mean age of 44.5 years (SD = 14.7). The majority (66.9%) were aged between 30 and 59 years, and 37.3% held a higher education degree. Sociodemographic profiles varied significantly according to relationship type. Immediate family members were predominantly women (70.2%), while distant family were mostly male (64.3%). Full demographic details are summarized in Table 1.

**TABLE 1:** Sociodemographic characteristics of participants from a multicentre study, categorized by type of relationship to patients with CSU (2023–2024).

		Relationship to patient										
Attribute	Description	Total		Immediate family		Extended relatives		Close relationships		Caregivers/healthcare providers		*p*‐value*
		*N*	%	*N*	%	*N*	%	*N*	%	*N*	%	
Gender	Female	682	57.5	466	70.2	154	35.7	52	68.4	10	62.5	<0.001*
	Male	505	42.5	198	29.8	277	64.3	24	31.6	6	37.5	
Age class	Less than 30 years	215	18.1	139	20.9	41	9.5	27	35.5	8	50.0	<0.001**
	30–59 years	794	66.9	439	66.1	310	72.1	37	48.7	8	50.0	
	60 years or older	177	14.9	86	13.0	79	18.4	12	15.8	0	0.0	
Ethnic group	African	10	0.8	6	0.9	4	0.9	0	0.0	0	0.0	<0.001**
	Asian	193	16.3	111	16.7	79	18.3	3	3.9	0	0.0	
	Caucasian	528	44.5	311	46.8	174	40.4	28	36.8	15	93.8	
	Latino/Hispanic	61	5.1	29	4.4	23	5.3	8	10.5	1	6.3	
	Middle Eastern	395	33.3	207	31.2	151	35.0	37	48.7	0	0.0	
Country	Brazil	119	10.0	64	9.6	50	11.6	3	3.9	2	12.5	<0.001**
	China	53	4.5	36	5.4	16	3.7	1	1.3	0	0.0	
	Ecuador	34	2.9	13	2.0	14	3.2	7	9.2	0	0.0	
	Greece	52	4.4	48	7.2	1	0.2	3	3.9	0	0.0	
	India	46	3.9	11	1.7	21	4.9	0	0.0	14	87.5	
	Oman	92	7.8	36	5.4	34	7.9	22	28.9	0	0.0	
	Perú	1	0.1	0	0.0	0	0.0	1	1.3	0	0.0	
	Poland	189	15.9	101	15.2	76	17.6	12	15.8	0	0.0	
	Russia	54	4.5	36	5.4	18	4.2	0	0.0	0	0.0	
	Thailand	50	4.2	27	4.1	21	4.9	2	2.6	0	0.0	
	Turkey	404	34.0	245	36.9	141	32.7	18	23.7	0	0.0	
	Macedonia	93	7.8	47	7.1	39	9.0	7	9.2	0	0.0	
Education level	No education	32	2.7	21	3.2	11	2.6	0	0.0	0	0.0	0.01**
	Elementary education	146	12.5	86	13.1	52	12.3	8	11.0	0	0.0	
	High school	295	25.3	159	24.3	117	27.7	11	15.1	8	53.3	
	Technical/vocational Education	114	9.8	69	10.5	37	8.7	7	9.6	1	6.7	
	Higher education	435	37.3	249	38.0	140	33.1	40	54.8	6	40.0	
	Postgraduate education	144	12.3	71	10.8	66	15.6	7	9.6	0	0.0	
Marital status	Common‐law marriage	37	3.2	22	3.4	14	3.3	1	1.4	0	0.0	<0.001**
	Divorced	37	3.2	29	4.4	5	1.2	1	1.4	2	13.3	
	Married	806	69.2	392	59.9	382	90.3	27	37.0	5	33.3	
	Single	268	23.0	198	30.3	19	4.5	44	60.3	7	46.7	

*Note*: *immediate family* (parents, siblings, children); *extended relatives* (spouse/partner, in‐laws); *close relationships* (grandparents, cousins, roommates/romantic partners); *caregivers* (live‐in caregivers providing daily assistance).

* Pearson's chi‐squared test.

** Fisher's exact test.

### FDLQI scores

**FIGURE 1:**
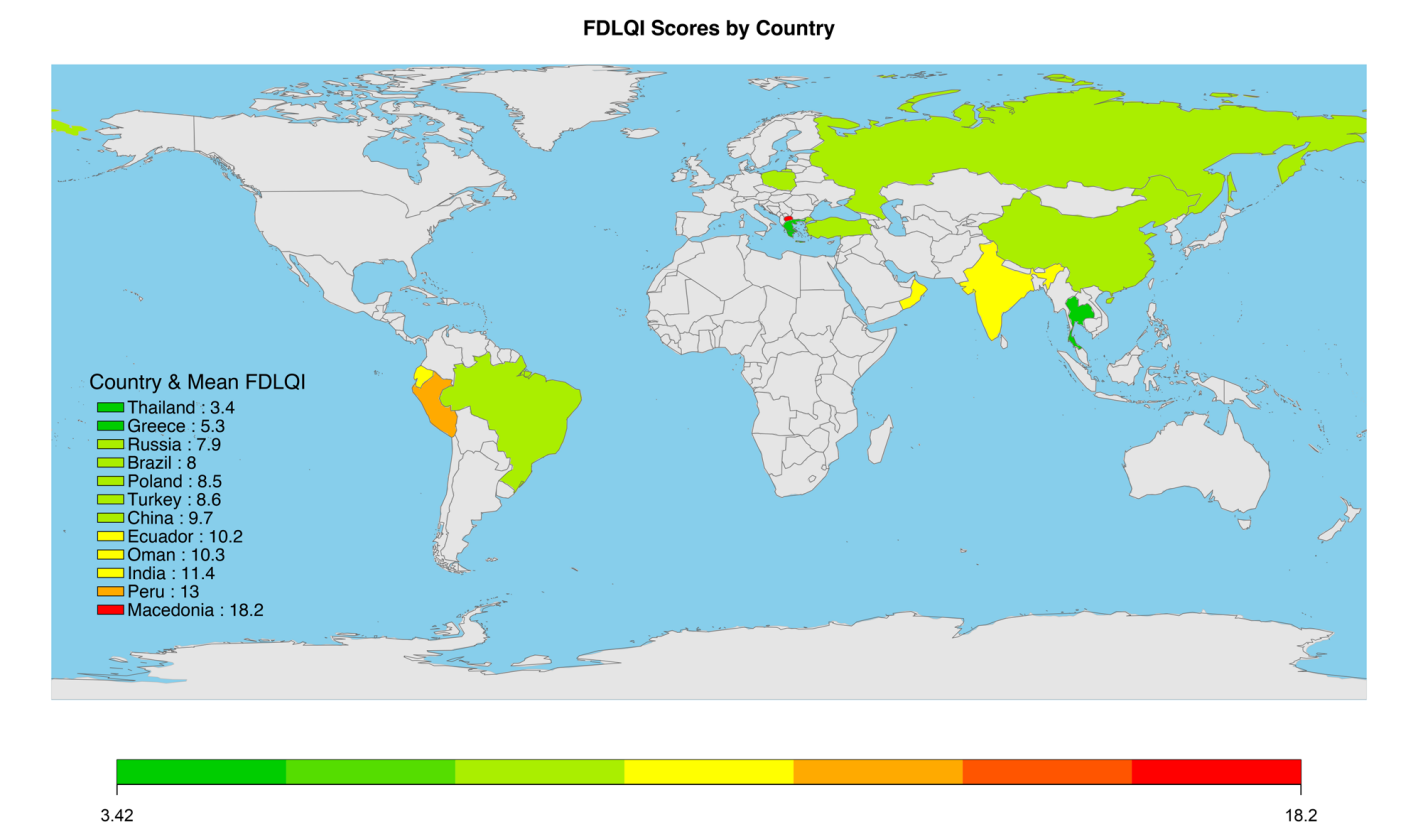
Distribution of the FDQLI mean scores by country (2023–2024).

FDLQI had a mean total score of 9.6 and mean scores varied across countries (Figure 1, Table S1). The lowest scores were observed in Thailand (3.42; 95% CI: 1.92–4.92) and Greece (5.35; 95% CI: 4.13–6.57). The highest impact was reported in North Macedonia (18.20; 95% CI: 16.97–19.44).

### CSU severity and control (UAS7 and UCT scores)

**FIGURE 2:**
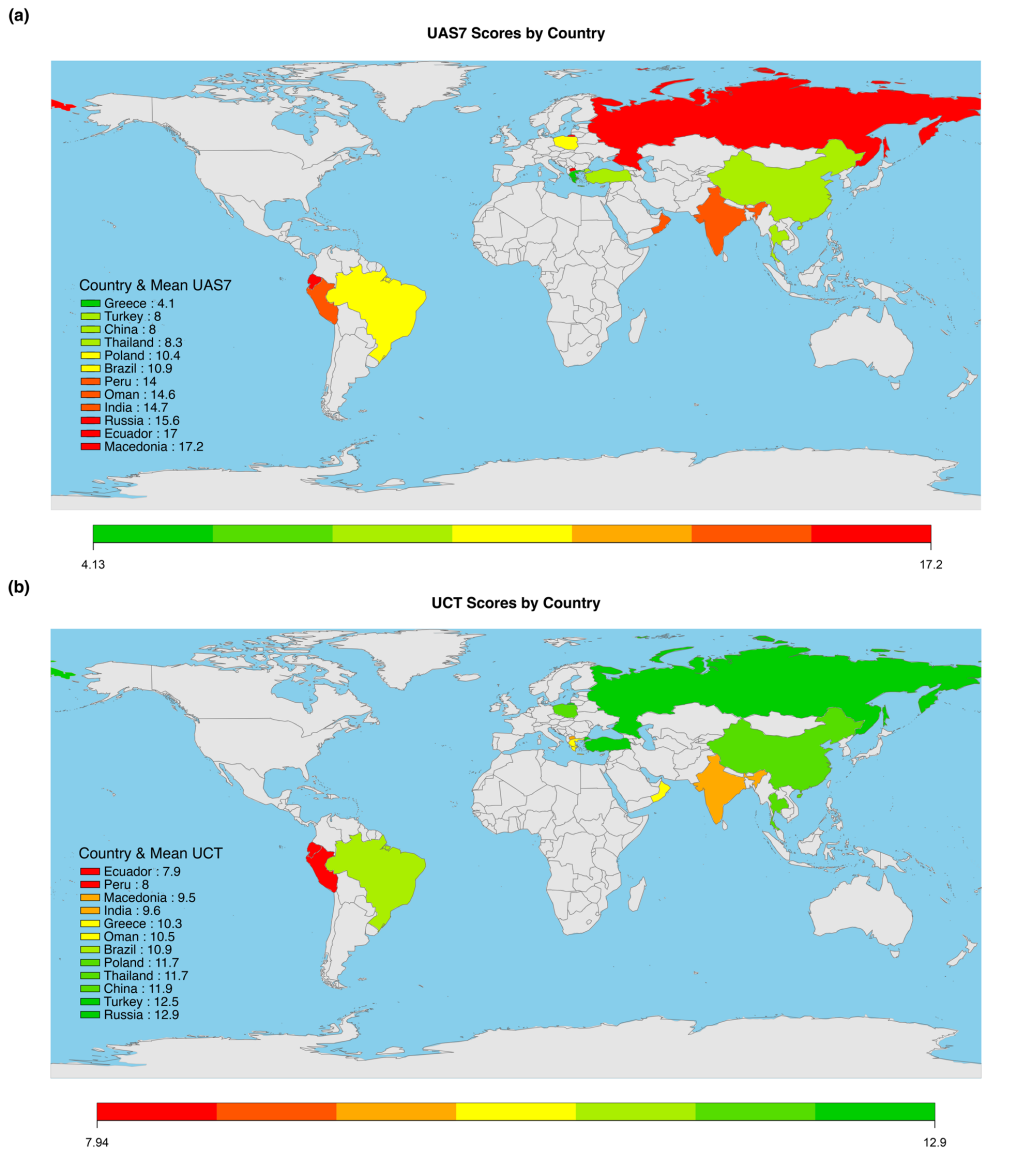
Distribution of the mean UAS7 (a) and UCT (b) scores by country (2023–2024).

CSU disease activity, measured by UAS7, showed wide geographic variation (Figure 2a, Table S1). Mean UAS7 scores were lowest in Greece (4.13; 95% CI: 3.24–5.03) and Turkey (7.96; 95% CI: 6.97–8.96). The highest scores were observed in North Macedonia (17.22; 95% CI: 15.18–19.25) and Ecuador (17.00; 95% CI: 11.76–22.24).

UCT scores varied across countries (Figure 2b). Higher scores were seen in Russia (12.93; 95% CI: 12.24–13.61), Turkey (12.50; 95% CI: 12.09–12.90) and Poland (11.65; 95% CI: 11.01–12.29). Conversely, Ecuador (7.94; 95% CI: 6.28–9.61), North Macedonia (9.53; 95% CI: 8.78–10.27) and India (9.61; 95% CI: 8.83–10.38) had low scores.

### Family QoL impact

A clear inverse association was observed between CSU control and family QoL across all FDLQI dimensions.

**FIGURE 3:**
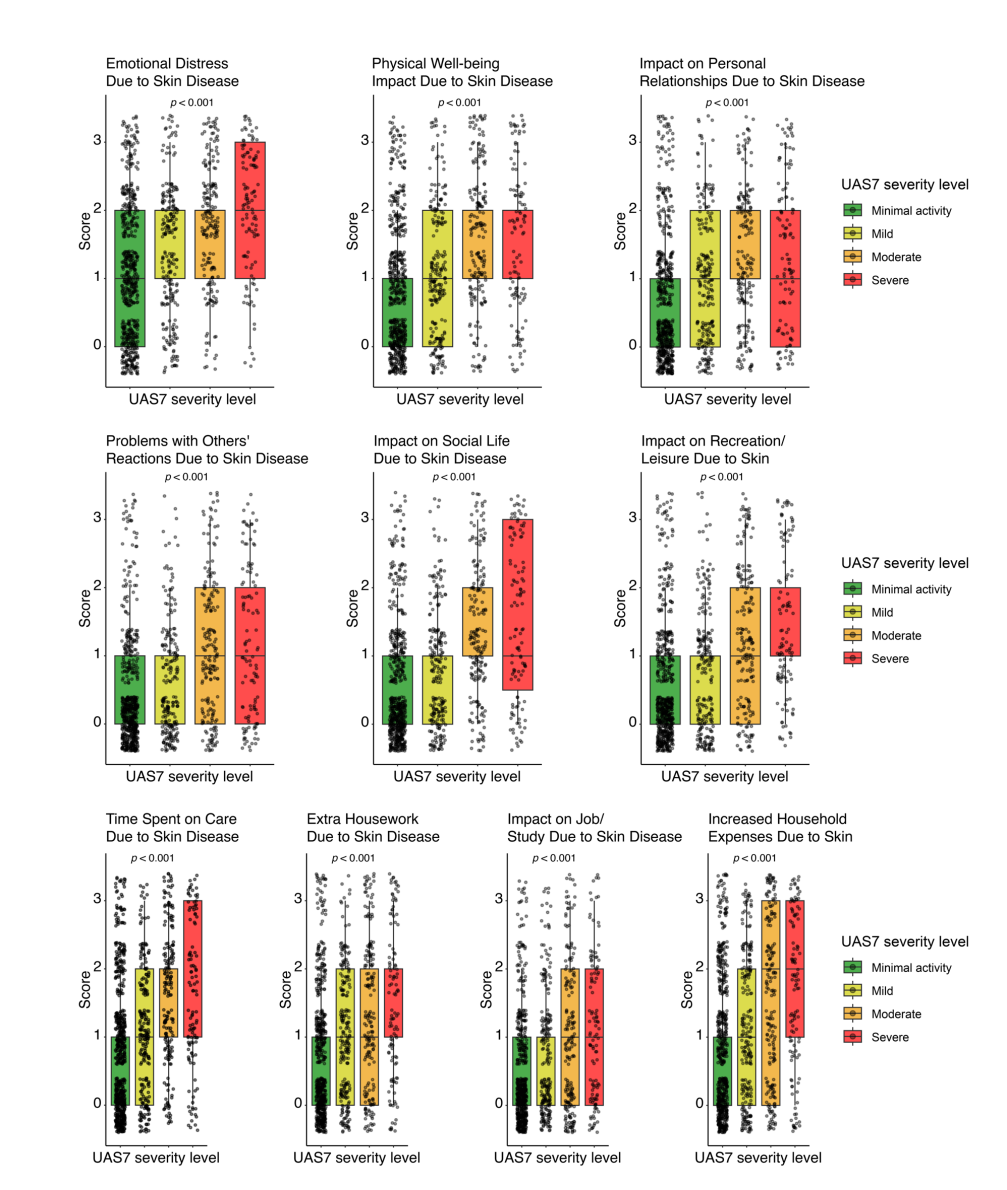
Comparison of FDLQI dimensions based on the levels of control of CSU assessed by UAS7. *p*‐value = Kruskal–Wallis; UAS7 scores: Minimal activity (UAS7 1–6), mild activity (UAS7 7–15), moderate activity (UAS7 16–27) and severe activity (UAS7 29–42).

When stratified by UAS7 severity (minimal activity, mild, moderate, severe), family members of patients with higher disease activity reported significantly greater emotional distress, physical burden, social limitations and financial strain (Figure 3, Table S2). Emotional distress was especially pronounced, with mean scores rising from 1.03 (SD = 0.96) in the minimal activity group to 2.04 (SD = 0.91) in the severe group (*p* < 0.001). Physical well‐being, social life impact and household expenses followed similar trends.

**FIGURE 4:**
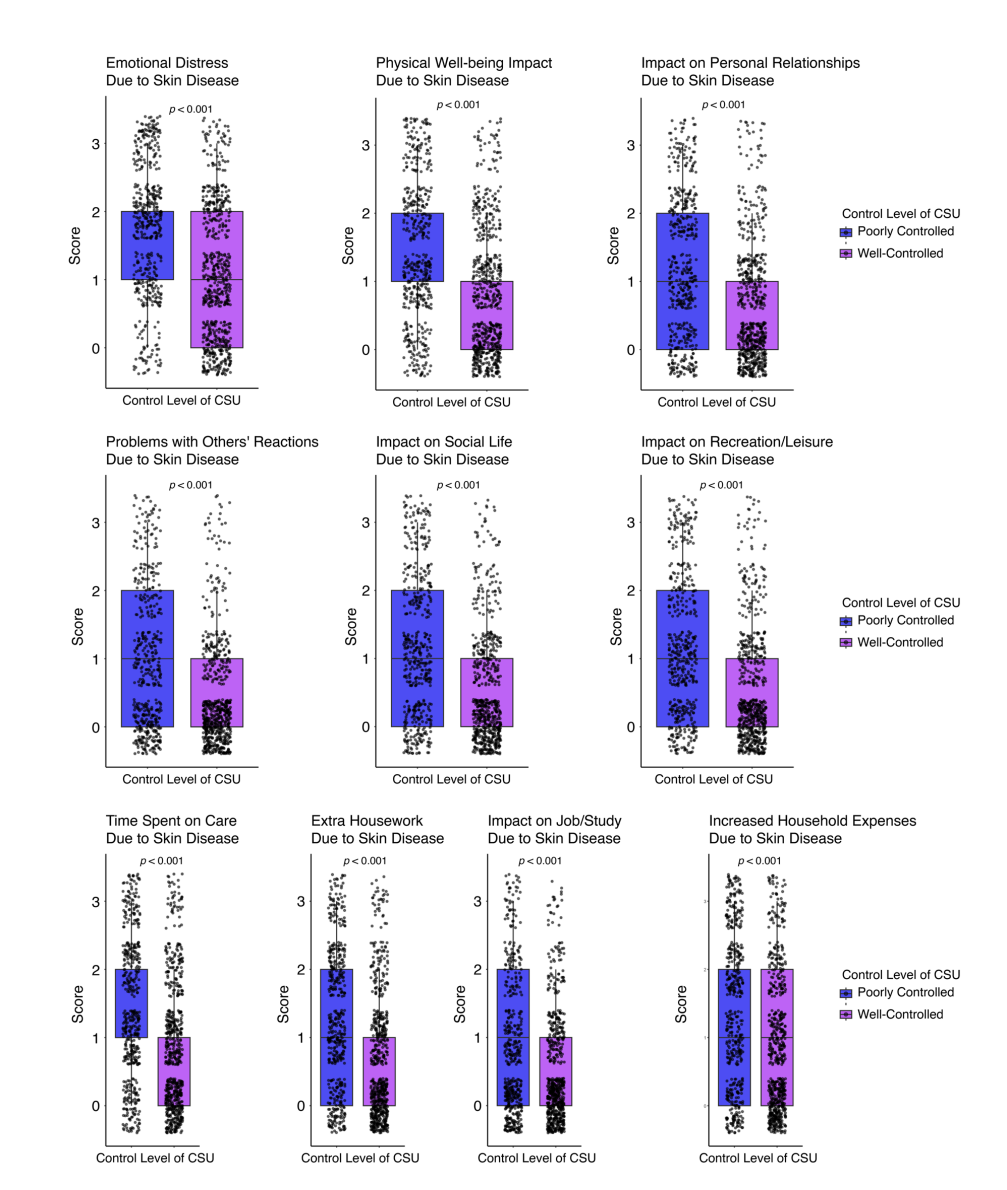
Comparison of FDLQI dimensions according to levels of control of CSU assessed by UCT.

Analysis using UCT categories (poorly controlled vs. well‐controlled) confirmed these findings (Figure 4, Table S3). Family members of poorly controlled CSU patients experienced higher emotional distress (1.75 ± 0.93 vs. 1.06 ± 0.95, p < 0.001), greater physical impact, more difficulties in interpersonal relationships and higher financial burden.

### Factors influencing family QoL (regression analysis results)

**FIGURE 5:**
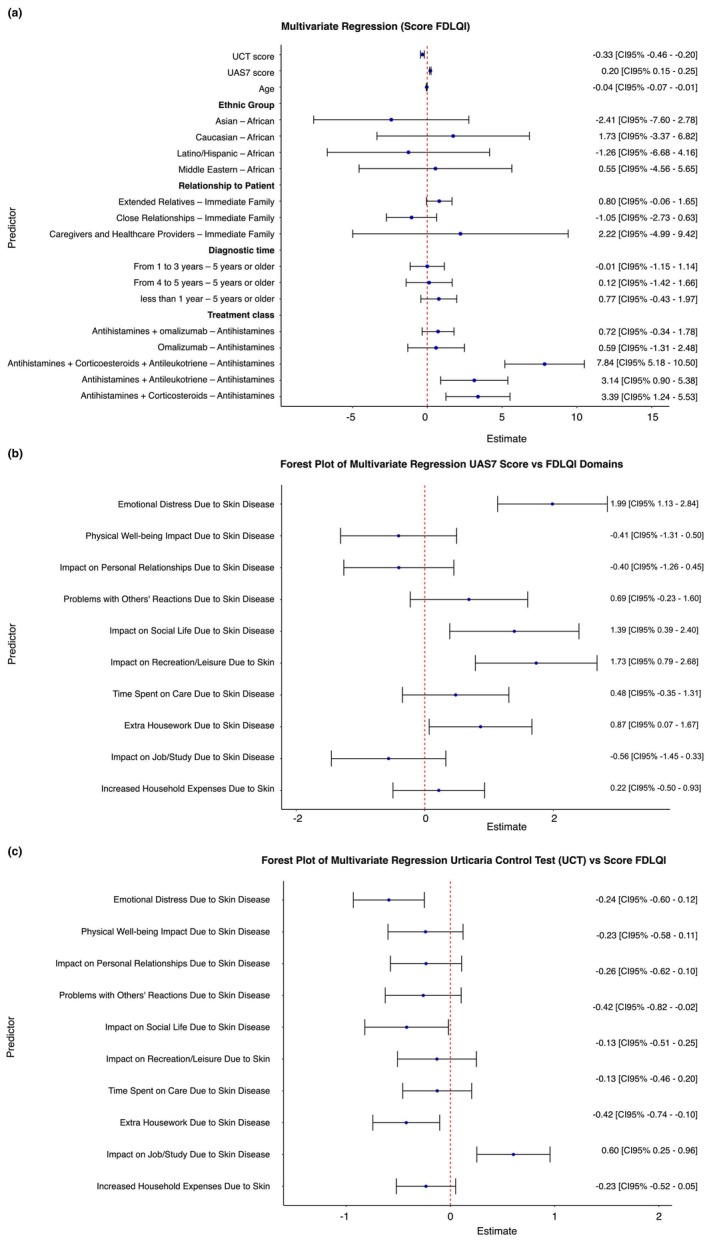
(a) Multivariate regression analysis of UAS7 and UCT in relation to FDLQI; (b) association between UAS7 and the different domains of the FDLQI in a multivariate regression analysis; (c) association between UCT and the different domains of the FDLQI in a multivariate regression analysis.

Results (Figure 5a) indicated that better CSU control was significantly associated with improved family QoL. Among demographic characteristics, older individuals reported less QoL impact (*β* = −0.04, CI95% –0.07 to −0.01); ethnicity showed no significant association; and close family relationships showed experienced lower QoL impact (*β* = −1.05; CI95% –2.73 to −0.63), with strong family bonds mitigating disease impact. Treatment regimen emerged as a significant factor in family QoL. The greatest burden was observed in those receiving antihistamines combined with corticosteroids and antileukotriene (*β* = 7.84; CI95% 5.18 to 10.50), while omalizumab (monotherapy) presented no significant association (*β* = 0.59; CI95% –1.31 to −2.48).

CSU severity affected emotional distress, social interactions and leisure activities (Figure 5b). Inadequate CSU management increased daily responsibilities for family members, such as time dedicated to patient care (0.53; CI95% –0.33 to 1.39) and additional household responsibilities (*β* = 0.76; CI95% –0.07 to 1.59), although confidence intervals still include a null value. Participation in professional and academic activities declined (*β* = −0.42; CI95% –1.53 to 0.49).

Greater efficacy in controlling CSU (Figure 5c) was associated with a lower impact on family members' QoL, particularly with emotional distress (*β* = −0.59; CI95% –0.93 to −0.25) and social life (*β* = −0.42; CI95% –0.82 to −0.02). Uncontrolled CSU increased additional housework (*β* = −0.42; CI95% –0.74 to −0.10). Conversely, better control significantly improved the participation in work or academic activities (*β* = 0.60; CI95% 0.25 to 0.96).

Household expenses showed no statistically significant association with either severity (*β* = 0.27; CI95% –0.47 to 1.00) or control (*β* = −0.23; CI95% –0.53 to 0.06).

### FDLQI in different skin diseases

**FIGURE 6:**
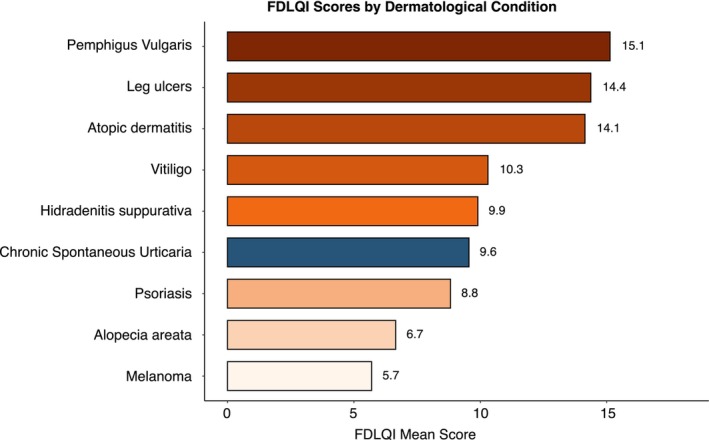
Comparison of mean FDLQI in different dermatological conditions.

FDLQI mean score for CSU was 9.6 and was compared to other skin diseases that have used this tool (Figure 6).^17^, ^31^, ^32^, ^33^, ^34^, ^35^, ^36^, ^37^, ^38^, ^39^ In CSU, the most affected FDLQI domains were emotional distress, social life limitations and recreational or leisure restriction, particularly among family members of patients with severe or poorly controlled disease. Emotional distress scores rose from 1.03 in families of patients with minimal activity to 2.04 in those with severe activity, while social life impact scores increased from 0.88 to 2.23, and physical burden and interpersonal relationship scores followed similar trends.

In leg ulcers, the total mean FDLQI was 14.4, with over 96% of caregivers reporting impaired QoL; however, this burden was largely attributed to physical caregiving, mobility constraints and household disruption.^37^ In pemphigus vulgaris (PV), mean FDLQI was 15.1, with high scores reported in emotional, caregiving and sleep disturbance domains, but less financial or social role impact than in CSU.^19^ For psoriasis, mean score was lower (8.8), and although associated with increased anxiety and depression, social and recreational interference scores were less pronounced compared to CSU.^36^ In alopecia areata, FDLQI scored 6.7, with highest impact in the emotional domain, particularly for younger children or more severe subtypes (alopecia totalis or universalis).^38^

Similarly, vitiligo family members reported mean FDLQI of 10.3, with emotional impact and appearance‐related stigma as dominant issues, yet recreational and household role scores remained lower than those observed in CSU.^17^ In hidradenitis suppurativa, the mean FDLQI of 9.9 reflected a broad burden across physical, emotional and sexual well‐being, though CSU families reported greater limitation in social life and daily participation.^31^, ^32^ In AD, mean FDLQI was 14.1, where sleep disruption and care complexity were high, yet again with lower social restriction scores than observed in moderate‐to‐severe CSU.^35^

## DISCUSSION

This international multicentre study indicated that CSU significantly impacts not only patients but also their family members. Furthermore, the study showed that better CSU management could substantially reduce family burden.

Similar results were identified by Gonçalo et al.,^10^ who highlighted the significant economic and social burden of CSU, particularly when accompanied by angioedema. The literature consistently highlights the need to view dermatological diseases comprehensively, considering not only physical symptoms but also their psychological and social effects.^3^, ^25^

International variations observed in CSU burden underscore the importance of cultural and socio‐economic differences in disease perception and management. Meeuwesen et al.^26^ emphasized that cultural dimensions directly influence medical communication and the perception of chronic illnesses, while Markus et al.^27^ pointed out cultures differ in how they address emotional and social aspects of disease. Additionally, patient journeys may differ due to different healthcare systems across countries affecting time of diagnosis and intervention.^40^, ^41^ These cultural factors likely contributed to regional variations, especially when contrasting countries with high family impact, such as North Macedonia, to those with lower impact, such as Thailand and China.

An important finding was the strong association between poor disease control and greater emotional and social burden on family members, supporting previous studies that evaluated the impact of other chronic dermatoses using FDLQI. Studies on HS, PV, AD, psoriasis, leg ulcers, AA, vitiligo and melanoma reported similar findings, highlighting emotional burden in these chronic dermatoses.^17^, ^31^, ^32^, ^33^, ^34^, ^35^, ^36^, ^37^, ^38^, ^39^ Our study is the first to assess CSU in this context, revealing a higher negative impact on FDLQI than melanoma, alopecia areata and psoriasis. PV patients' family members reported marked emotional and physical burden, particularly in cases of mucocutaneous involvement, though financial impact was less emphasized than in CSU.^19^ In AD, caregiver QoL was significantly impaired by disease severity and communication barriers, but the condition's predictability contrasts with the fluctuating, uncontrollable nature of CSU.^35^ In psoriasis FDQLI levels were comparable to the psoriasis patients themselves, though were lower than those observed in CSU families.^36^ HS had a high QoL impact, with surgery and adalimumab improving outcomes.^32^ However, partners of HS patients also faced substantial sexual dysfunction and psychological strain, showing overlap with CSU's emotional and social effects, though CSU showed more widespread interference in daily responsibilities.^31^

Socio‐economic aspects, particularly increased management costs associated with poor disease management, remain overlooked in clinical studies. Gonçalo et al.^10^ noted CSU can generate substantial expenses, especially in cases involving prolonged and poorly managed treatment, resulting in a significant family economic burden. Augustin et al.^42^ showed disease control was strongly associated with reduced productivity loss, highlighting the need for more effective treatments. The study showed around 26% work productivity impairment and 31% activity impairment, contributing to around 2.2‐billion‐euro annual socio‐economic burden, two‐thirds due to unpaid work.^42^

Recent studies on CSU pathophysiology also help explain the challenges in achieving effective clinical control. Kaplan et al.^25^ and Hide et al.^22^ highlight that the complexity of the immunological mechanisms underlying CSU, including autoimmunity and exacerbated mast cell activation, may partly explain difficulties in achieving adequate clinical control. These factors underscore the need for personalized care and novel therapies targeting immune pathways, as suggested by Kulthanan et al.^43^ Disease control was strongly correlated with reduced productivity loss, underscoring the need for more effective treatments and healthcare strategies.

These findings highlight the value of an interdisciplinary approach to managing CSU, where educational and psychosocial interventions could have a role in complementing pharmacological therapies. Tomaszewska et al.^44^ suggest that psychosocial and educational interventions significantly improve disease control and prolong remission periods, reducing overall family burden. Thus, in addition to traditional pharmacological control, complementary strategies should be routinely considered in clinical practice to minimize CSU impact on patients and families, effectively contributing to improved QoL within a broader family context.

Our study stands out for its internationally diverse sample and for the use of validated and standardized instruments to assess CSU impact on family members. The geographic diversity of participants allowed for the observation of the influence of cultural and structural aspects on disease experience, while multivariate analysis enabled the identification of independent factors associated with higher or lower family burden. Furthermore, the association between disease control and family QoL supports its practical value in guiding care strategies.

The multivariate regression analysis provided insights into specific demographic predictors of family impact. Older individuals reported less impact, possibly due to effective coping and adaptation strategies over time.^39^, ^45^ Immediate family members were less affected than distant family members, suggesting the protective role of closer familial bonds in chronic disease. Goldstein et al.^46^ highlighted that while some patients with CSU reported consistent empathy and encouragement from close family, others experienced relational strain and perceived themselves as a burden, with support diminishing over time. This variability reinforces the potential buffering effect of immediate family proximity in reducing psychosocial distress.

An important finding was the relationship between treatment type and family impact, where more complex regimens were directly associated with greater negative QoL impact. This aligns with the review by Kolkhir et al.,^3^ who reported that stepped and complex treatments can heighten emotional and physical stress experienced by patients and their families due to administration complexity and associated side effects. On the other hand, the isolated or combined use of omalizumab showed no significant negative impact, confirming the safety and efficacy as a recommended therapeutic strategy.^24^, ^47^

The treatment dimension also warrants further exploration in the context of family burden. Omalizumab has shown efficacy in both clinical trials and real‐world settings, reducing disease severity, enhancing symptom management and QoL.^48^ It is well‐tolerated in CSU, with discontinuation most often related to disease control rather than adverse events or ineffectiveness.^49^ A large international cohort, nearly half of patients had longstanding CSU, and 77% required higher‐than‐standard dosing, underscoring the need for individualized treatment strategies.^49^ Drug survival was longer in fast responders, while discontinuation due to inefficacy was associated with autoimmune comorbidities and baseline immunosuppressive use.^49^ However, a recent study found that despite treatment, 32.9% of participants experienced no change in their QoL, while 35.7% reported mild improvement and 31.4% had moderate improvement, suggesting that variability in response may be attributed to differences in specific aspects of the disease.^48^ These findings align with guideline recommendations placing omalizumab after high‐dose antihistamines and before cyclosporine, though up to one‐third of patients may still show partial or no response.^3^ Simplified and effective regimens like omalizumab may help reduce the emotional and logistical burden on families, particularly compared to more complex therapeutic strategies.

Among the limitations, we acknowledge that the cross‐sectional design does not allow for causal inference. Although temporal relationships between variables cannot be established, the findings may be considered hypothesis‐generating and provide a basis for future longitudinal or interventional studies. Additionally, although the FDLQI is validated for dermatological conditions, there is no evidence of its prior application in CSU. Therefore, while our findings offer important insights, they should be considered as preliminary subject to further evaluation. Also, potential selection bias is worth regarding, as participants were recruited from UCARE centres and participation was voluntary, possibly leading to overrepresentation of more engaged or highly educated individuals. However, the internal consistency of the results, the statistical strength of the sample, and the alignment with previous studies lend robustness to the conclusions. These findings emphasize the need to include CSU patients' families in healthcare strategies, particularly through emotional support, psychosocial monitoring and guidance aimed at reducing family suffering.

To conclude, this study highlights that CSU significantly burdens not only patients but also their families. The link between poor disease control and greater family impact emphasizes the need for effective, patient‐centred treatments. Integrating family support and health education into care strategies is essential to improve QoL across the household.

## AUTHOR CONTRIBUTIONS

Conceptualization: BMZA, RFJC, KK, EK, MICO, AMGA, MM, LFE, JB, MH and PRC; Methodology: BMZA, RFJC and PRC; Data collection: BMZA, RFJC, KK MICO, LFE, SDDJ, SORV, GD, PX, PD, IP, VT, NTM, IHN, BAH, JB, DK, PSK, HG, DO, KC, EO, YE, ME, MT, ROK, JL, AA and PRC.; Formal analysis: BMZA, RFJC, EK and PRC.; Writing—original draft preparation: BMZA.; writing—review and editing: RFJC, EK and PRC.; Supervision: PRC.

## FUNDING INFORMATION

None.

## CONFLICT OF INTEREST STATEMENT

BMZA, KK, EK, ICO, MM, LFE, JB, MH, PX, PD, NTM, IHN, BAH, DK, HG, DO, KC, YE, ME, JL, AA and MT declare no conflicts of interest related to this manuscript. RFJC reports consulting fees and honoraria for lectures from Novartis, Pfizer, AbbVie and Sanofi, and travel support from Pfizer and Sanofi. AMGA has received research support from ESCIENT, NOUCOR, Novartis, Instituto Carlos III–FEDER and Uriach Pharma; consulting fees from Almirall, Genentech, GSK, Novartis, Sanofi–Regeneron and others; honoraria for lectures from Avene, MSD, Menarini and additional companies; travel support from Novartis; and has served on the advisory board for Celldex. JAB has served as investigator and consultant at ADARx, Ajou University, Allergy therapeutics, Amgen, Apogee, Areteia, ARS, Astra Zeneca, Astria, Biocyrst, Blueprint Medicine, Celldex, Cogent, CSL Behring, Eli Lilly, Escient, Evommune, Fresenius Kabi, Genentech, GSK, Incyte, Intellia, Ionis, Japan Tobacco Company, Jasper, Kalvista, Kenvue, Kymeria, Kyowa Kirin, Medscape, Merck, Nasus, Neffy, Nektar, Neopharma, Novartis, Opella, Pharming, Pharvaris, Proctor and Gamble, Regeneron, Sanofi, Takeda/Shire, Telios, Teledoc, TEVA, Yuhan and WebMD news. He is a consultant at Enanta, Pfizer, RAPT and speaker at Pharming, Kalvista and CSL Pharming. MH has received consulting fees and lecture honoraria from Novartis, Sanofi, Japan Tobacco, and various Japanese pharmaceutical companies, and travel support from TAIHO Pharmaceutical. SDDJ reports consulting fees and honoraria from AstraZeneca, GSK, Novartis, Sanofi and Glenmark. SORV reports consulting fees, honoraria, travel support and advisory board participation with Novartis and Sanofi. GACD reports honoraria from Novartis and Sanofi, and travel support from Sanofi. VT reports honoraria for lectures from Uriach and Berlin‐Chemie Menarini. JB reports honoraria from Medac, Novartis, Galderma, and Pierre Fabre. EO reports institutional consulting fees from Sanofi‐Genzyme and Pfizer; and served as principal investigator for clinical trials with Amgen, Novartis, and Sanofi Genzyme; she also holds unpaid academic roles with Contact Dermatitis (Wiley), ESCD and EADV. MT reports similar trial leadership and consulting fees paid to his institution from Amgen, Novartis and Sanofi Genzyme; and holds unpaid roles on editorial boards and scientific committees. ROK reports honoraria for lectures from Menarini, Lilly, AbbVie, Novartis and La Roche‐Posay. AA was a speaker for Novartis, outside the scope of this work. PRC reports consulting fees and honoraria for lectures from Novartis, Pfizer, AbbVie, Galderma and Sanofi, and travel support from Pfizer and Sanofi.

## ETHICAL APPROVAL

Reviewed and approved by Brazilian IRB approval: #4931797.

## ETHICS STATEMENT

The patients in this manuscript have given written informed consent to publication of their case details.

## Supporting information


Data S1.

## Data Availability

Data available in article supplementary material.
