# Supplementary material for: Family quality‐of‐life burden in chronic spontaneous urticaria: A multicentre study
Source: J Eur Acad Dermatol Venereol. 2026 Jan 5;40(8):1406–19. doi: 10.1111/jdv.70290 (PMC13425259; doi:10.1111/jdv.70290)
Supplement: Supplementary file 1 — Data S1. [file JDV-40-1406-s001.docx]

Supplementary Material

**Supplementary Methods**

The following section provides extended methodological details not included in the main manuscript due to space constraints. These include additional information on population and inclusion criteria, including ethical approvals, data collection and statistical analysis.

*Population and Inclusion Criteria*

All participants were provided with comprehensive information regarding the study´s objectives and procedures and were assured the confidentiality of their responses. Subsequently, each collaborating centre obtained study approval from their respective local Ethics Committee.

Permission to use the FDLQI use for this academic study was granted by Finley in Cardiff University (UK), accessible at <https://www.cardiff.ac.uk/medicine/resources/quality-of-life-questionnaires/family-dermatology-life-quality-index>.

*Data collection*

The FDLQI is a validated tool for assessing how dermatological conditions affect family members’ QoL. It consists of 10 questions covering emotional distress, physical well-being, personal relationships, challenges related to others’ reactions to the patient’s condition (societal reactions), social life, leisure and recreational activities, caregiving time, additional household tasks, work or study interferences, and disease-related household expenses. Each item is scored on a 4-point Likert scale from 0 (“not at all/not relevant”) to 3 (“very much”), yielding a total score from 0 to 30, where higher scores indicate greater QoL impairment.

UAS7 evaluates disease symptoms frequency and intensity over the preceding week, with higher scores reflecting higher disease activity. UCT assesses disease control over the past month, with higher scores indicating better control.

*Statistical Analysis*

To explore the spatial distribution of FDLQI scores, UAS7 (0 = no urticaria; 1–6 = under control; 7–15 = mild; 16–27 = moderate; 28–42 = severe), and UCT (<12 = uncontrolled; 12–15 = well-controlled; 16 = completely controlled) across the participating countries, maps were created to represent mean score variation by country. This approach facilitated the identification of geographic patterns in CSU’s impact on families.

Normality of FDLQI scores was assessed using the Kolmogorov-Smirnov test, which indicated a non-normal distribution. Accordingly, non-parametric methods were employed. Multiple comparisons following Kruskal-Wallis were applied where necessary.

Regression analyses were preceded by verification of key assumptions. Multicollinearity was assessed using the Variance Inflation Factor (VIF), showing no substantial issues. Residual normality was confirmed through Shapiro-Wilk tests and histograms, and homoscedasticity was verified via residual plots.

Graphs were generated using the ggplot2 package in R. Data collection was conducted via Google Forms®, ensuring anonymity and compliance with ethical and data protection standards.

**Supplementary Table 1.** Mean Scores and 95% Confidence Intervals for FDLQI, UAS7, and UCT

| **FDLQI score** | | | | **95% Confidence interval** | |  |
| --- | --- | --- | --- | --- | --- | --- |
|  | **Country** | **N** | **Mean** | **Lower** | **Upper** | **SD** |
|  | Brazil | 119 | 8.03 | 6.72 | 9.33 | 7.29 |
|  | China | 53 | 9.68 | 7.35 | 12.01 | 8.67 |
|  | Ecuador | 34 | 10.24 | 8.29 | 12.18 | 5.77 |
|  | Greece | 52 | 5.35 | 4.13 | 6.57 | 4.48 |
|  | India | 46 | 11.37 | 10.13 | 12.61 | 4.29 |
|  | Oman | 92 | 10.30 | 8.77 | 11.84 | 7.52 |
|  | Perú | 1 | 13.00 | -- | -- | -- |
|  | Poland | 189 | 8.54 | 7.51 | 9.57 | 7.20 |
|  | Russia | 54 | 7.91 | 6.28 | 9.53 | 6.10 |
|  | Thailand | 50 | 3.42 | 1.92 | 4.92 | 5.39 |
|  | Turkey | 404 | 8.56 | 7.88 | 9.23 | 6.90 |
|  | Macedonia | 93 | 18.20 | 16.97 | 19.44 | 6.06 |
| **UAS7 score** |  |  |  | **95% Confidence interval** | |  |
|  | **Country** | **N** | **Mean** | **Lower** | **Upper** | **SD** |
|  | Brazil | 108 | 10.88 | 8.50 | 13.26 | 12.62 |
|  | China | 53 | 7.98 | 5.77 | 10.20 | 8.23 |
|  | Ecuador | 34 | 17.00 | 11.76 | 22.24 | 15.60 |
|  | Greece | 52 | 4.13 | 3.24 | 5.03 | 3.28 |
|  | India | 46 | 14.74 | 12.05 | 17.43 | 9.30 |
|  | Oman | 92 | 14.59 | 12.36 | 16.81 | 10.90 |
|  | Perú | 1 | 14.00 | -- | -- | -- |
|  | Poland | 189 | 10.37 | 8.76 | 11.97 | 11.28 |
|  | Russia | 54 | 15.57 | 13.44 | 17.71 | 8.00 |
|  | Thailand | 50 | 8.26 | 5.70 | 10.82 | 9.25 |
|  | Turkey | 404 | 7.96 | 6.97 | 8.96 | 10.21 |
|  | Macedonia | 93 | 17.22 | 15.18 | 19.25 | 10.01 |
| **UCT score** |  |  |  | **95% Confidence interval** | |  |
|  | **Country** | **N** | **Mean** | **Lower** | **Upper** | **SD** |
|  | Brazil | 119 | 10.93 | 10.03 | 11.84 | 5.03 |
|  | China | 53 | 11.94 | 10.89 | 13.00 | 3.93 |
|  | Ecuador | 34 | 7.94 | 6.28 | 9.61 | 4.95 |
|  | Greece | 52 | 10.31 | 8.96 | 11.65 | 4.96 |
|  | India | 46 | 9.61 | 8.83 | 10.38 | 2.68 |
|  | Oman | 92 | 10.48 | 9.75 | 11.21 | 3.56 |
|  | Perú | 1 | 8.00 | -- | -- | -- |
|  | Poland | 189 | 11.65 | 11.01 | 12.29 | 4.49 |
|  | Russia | 54 | 12.93 | 12.24 | 13.61 | 2.56 |
|  | Thailand | 50 | 11.66 | 10.79 | 12.53 | 3.13 |
|  | Turkey | 404 | 12.50 | 12.09 | 12.90 | 4.14 |
|  | Macedonia | 93 | 9.53 | 8.78 | 10.27 | 3.67 |

**Supplementary Table 2.** Distribution and comparison of FDLQI dimensions based on the levels of severity of CSU assessed by UAS7

| **Urticaria Activity Score 7 (UAS7)** | **Group** | **N** | **Mean** | **SD** | **SE** | **p-value*** |
| --- | --- | --- | --- | --- | --- | --- |
| Emotional distress due to skin disease | Minimal activity | 595 | 1.05 | 0.97 | 0.04 | <0,001 |
|  | Mild | 253 | 1.40 | 0.92 | 0.06 |  |
|  | Moderate | 206 | 1.83 | 0.85 | 0.06 |  |
|  | Severe | 122 | 2.06 | 0.91 | 0.08 |  |
| Physical well-being impact due to skin disease | Minimal activity | 595 | 0.81 | 0.94 | 0.04 | <0,001 |
|  | Mild | 253 | 1.08 | 0.92 | 0.06 |  |
|  | Moderate | 206 | 1.62 | 0.96 | 0.07 |  |
|  | Severe | 122 | 1.54 | 1.05 | 0.10 |  |
| Impact on personal relationships due to skin disease | Minimal activity | 595 | 0.66 | 0.90 | 0.04 | <0,001 |
|  | Mild | 253 | 0.95 | 0.91 | 0.06 |  |
|  | Moderate | 206 | 1.30 | 0.95 | 0.07 |  |
|  | Severe | 122 | 1.41 | 1.11 | 0.10 |  |
| Problems with others' reactions due to skin disease | Minimal activity | 595 | 0.41 | 0.76 | 0.03 | <0,001 |
|  | Mild | 253 | 0.61 | 0.81 | 0.05 |  |
|  | Moderate | 206 | 1.12 | 0.99 | 0.07 |  |
|  | Severe | 122 | 1.23 | 1.12 | 0.10 |  |
| Impact on social life due to skin disease | Minimal activity | 595 | 0.54 | 0.83 | 0.03 | <0,001 |
|  | Mild | 253 | 0.79 | 0.85 | 0.06 |  |
|  | Moderate | 206 | 1.36 | 0.97 | 0.07 |  |
|  | Severe | 122 | 1.50 | 1.14 | 0.11 |  |
| Impact on recreation/leisure due to skin disease | Minimal activity | 595 | 0.55 | 0.85 | 0.04 | <0,001 |
|  | Mild | 253 | 0.78 | 0.85 | 0.06 |  |
|  | Moderate | 206 | 1.29 | 1.03 | 0.07 |  |
|  | Severe | 122 | 1.50 | 1.05 | 0.10 |  |
| Time spent on care due to skin disease | Minimal activity | 595 | 0.80 | 0.93 | 0.04 | <0,001 |
|  | Mild | 253 | 1.06 | 0.90 | 0.06 |  |
|  | Moderate | 206 | 1.62 | 0.97 | 0.07 |  |
|  | Severe | 122 | 1.60 | 1.06 | 0.10 |  |
| Extra housework due to skin disease | Minimal activity | 595 | 0.68 | 0.90 | 0.04 | <0,001 |
|  | Mild | 253 | 1.01 | 0.90 | 0.06 |  |
|  | Moderate | 206 | 1.34 | 1.04 | 0.08 |  |
|  | Severe | 122 | 1.50 | 1.09 | 0.10 |  |
| Impact on job/study due to skin disease | Minimal activity | 595 | 0.43 | 0.75 | 0.03 | <0,001 |
|  | Mild | 253 | 0.71 | 0.89 | 0.06 |  |
|  | Moderate | 206 | 0.97 | 1.03 | 0.07 |  |
|  | Severe | 122 | 1.08 | 1.04 | 0.10 |  |
| Increased household expenses due to skin disease | Minimal activity | 595 | 0.82 | 0.97 | 0.04 | <0,001 |
|  | Mild | 253 | 1.13 | 1.02 | 0.07 |  |
|  | Moderate | 206 | 1.50 | 1.15 | 0.08 |  |
|  | Severe | 122 | 1.64 | 1.13 | 0.10 |  |

**Note:** SD: Standard Deviation; SE: Standard Error; *Kruskal Wallis; Group UAS7 scores: Minimal activity (UAS7 1-6), Mild activity (UAS7 7-15), Moderate activity (UAS7 16-27), Severe activity (UAS7 29-42)

**Supplementary Table 3.** Distribution and comparison of FDLQI dimensions based on the levels of control of CSU assessed by UCT

| **Urticaria Control Test (UCT)** | **Group** | **N** | **Mean** | **SD** | **SE** | **p-value*** |
| --- | --- | --- | --- | --- | --- | --- |
| Emotional distress due to skin disease | Poorly controlled | 494 | 1.75 | 0.93 | 0.04 | <0,001 |
|  | Well-controlled | 693 | 1.06 | 0.95 | 0.04 |  |
| Physical well-being impact due to skin disease | Poorly controlled | 494 | 1.44 | 1.01 | 0.05 | <0,001 |
|  | Well-controlled | 693 | 0.81 | 0.92 | 0.04 |  |
| Impact on personal relationships due to skin disease | Poorly controlled | 494 | 1.27 | 1.03 | 0.05 | <0,001 |
|  | Well-controlled | 693 | 0.63 | 0.84 | 0.03 |  |
| Problems with others' reactions due to skin disease | Poorly controlled | 494 | 0.95 | 1.01 | 0.05 | <0,001 |
|  | Well-controlled | 693 | 0.42 | 0.75 | 0.03 |  |
| Impact on social life due to skin disease | Poorly controlled | 494 | 1.19 | 1.03 | 0.05 | <0,001 |
|  | Well-controlled | 693 | 0.55 | 0.81 | 0.03 |  |
| Impact on recreation/leisure due to skin | Poorly controlled | 494 | 1.18 | 1.03 | 0.05 | <0,001 |
|  | Well-controlled | 693 | 0.55 | 0.82 | 0.03 |  |
| Time spent on care due to skin disease | Poorly controlled | 494 | 1.40 | 1.01 | 0.05 | <0,001 |
|  | Well-controlled | 693 | 0.83 | 0.93 | 0.04 |  |
| Extra housework due to skin disease | Poorly controlled | 494 | 1.30 | 1.01 | 0.05 | <0,001 |
|  | Well-controlled | 693 | 0.68 | 0.88 | 0.03 |  |
| Impact on job/study due to skin disease | Poorly controlled | 494 | 0.87 | 1.00 | 0.05 | <0,001 |
|  | Well-controlled | 693 | 0.49 | 0.78 | 0.03 |  |
| Increased household expenses due to skin | Poorly controlled | 494 | 1.41 | 1.11 | 0.05 | <0,001 |
|  | Well-controlled | 693 | 0.85 | 0.99 | 0.04 |  |

**Note:** SD: Standard Deviation; SE: Standard Error; *Mann Whitney U
